# A large new subset of TRIM genes highly diversified by duplication and positive selection in teleost fish

**DOI:** 10.1186/1741-7007-7-7

**Published:** 2009-02-05

**Authors:** Lieke M van der Aa, Jean-Pierre Levraud, Malika Yahmi, Emilie Lauret, Valérie Briolat, Philippe Herbomel, Abdenour Benmansour, Pierre Boudinot

**Affiliations:** 1 Virologie et Immunologie Moléculaires, Institut National de la Recherche Agronomique, Jouy-en-Josas, France; 2 Cell Biology and Immunology Group, Wageningen University, Marijkeweg 40, 6709 PG, Wageningen, The Netherlands; 3 Unité Macrophages et Développement de l'Immunité, URA 2578 du Centre National de la Recherche Scientifique, Institut Pasteur, 25, rue du Docteur Roux, 75015 Paris, France

## Abstract

**Background:**

In mammals, the members of the tripartite motif (TRIM) protein family are involved in various cellular processes including innate immunity against viral infection. Viruses exert strong selective pressures on the defense system. Accordingly, antiviral TRIMs have diversified highly through gene expansion, positive selection and alternative splicing. Characterizing immune TRIMs in other vertebrates may enlighten their complex evolution.

**Results:**

We describe here a large new subfamily of TRIMs in teleosts, called finTRIMs, identified in rainbow trout as virus-induced transcripts. FinTRIMs are formed of nearly identical RING/B-box regions and C-termini of variable length; the long variants include a B30.2 domain. The zebrafish genome harbors a striking diversity of finTRIMs, with 84 genes distributed in clusters on different chromosomes. A phylogenetic analysis revealed different subsets suggesting lineage-specific diversification events. Accordingly, the number of *fintrim *genes varies greatly among fish species. Conserved syntenies were observed only for the oldest *fintrims*. The closest mammalian relatives are *trim16 *and *trim25*, but they are not true orthologs. The B30.2 domain of zebrafish finTRIMs evolved under strong positive selection. The positions under positive selection are remarkably congruent in finTRIMs and in mammalian antiviral TRIM5α, concentrated within a viral recognition motif in mammals. The B30.2 domains most closely related to finTRIM are found among NOD-like receptors (NLR), indicating that the evolution of TRIMs and NLRs was intertwined by exon shuffling.

**Conclusion:**

The diversity, evolution, and features of finTRIMs suggest an important role in fish innate immunity; this would make them the first TRIMs involved in immunity identified outside mammals.

## Background

Newly discovered players in the antiviral immunity field are the proteins belonging to the tripartite motif (TRIM) family. The TRIM proteins are characterized by a tripartite motif that comprises from the N- to C-terminus, a RING zinc finger domain, one or two B-boxes and a coiled-coil domain. They are therefore also known as RBCC proteins [1]. The RING finger and B-box are cysteine-rich domains and both domains bind zinc atoms, suggesting interaction with other proteins, RNA and DNA [2-5]. They are usually encoded as a single exon, and together form the 'RBB' region. In addition, the RING finger has E3 ubiquitin ligase activity [6]. The coiled-coil region seems to be predominantly necessary for multimerization, resulting in the formation of high-molecular weight complexes. In many TRIM proteins an additional domain is present at the C-terminus [7], with the B30.2 domain being the most frequent one (reviewed in [8]). The B30.2 domain is encoded by one exon [9,10]. The domain is also found in butyrophilin and stonustoxin [11] and has evolved by a relatively recent juxtaposition of the PRY domain and the SPRY domain; it is therefore also known as the PRY/SPRY domain [12]. The B30.2 domain has been shown to be essential for ligand binding in several TRIM proteins [13-15]. Its tertiary structure has recently been elucidated for TRIM21, revealing two binding pockets formed by six variable loops [16]. Since the order and spacing of the domains are highly conserved, a TRIM protein presumably acts as an integrated structure [1]. TRIM proteins are evolutionarily old proteins that can be found in primitive metazoans [6]. Currently, 68 TRIM-encoding genes have been described in human [1,7,8,17]. Most TRIM genes code for at least two isoforms that are generated by alternative splicing, resulting in full-length and partial transcripts that lack the C-terminal encoding sequence.

The TRIM proteins play multiple roles in various cellular processes, which include cell growth, differentiation and apoptosis in mammals. Many TRIM genes are proto-oncogenes and severe diseases such as Opitz syndrome and acute promyelocytic leukemia are caused by mutations in *trim18 *and *trim19*, respectively [18], reviewed in [19]. An antiviral activity has also been described for several TRIM proteins: TRIM1, -5α, -11, -15, -19, -22, -25, -28 -32 [8,20-22]. These TRIM proteins can block viral infection by different mechanisms, as revealed by the functional characterization of TRIM5α, TRIM19 and TRIM25. A virus-specific interaction has been described for TRIM5α and TRIM19. TRIM5α was initially identified in rhesus macaques as the protein responsible for post-entry restriction of HIV-1 in this species, while its human ortholog could not block HIV-1 [23]. TRIM5α forms trimers that bind the nucleocapsid of incoming viral particles through a C-terminal B30.2 domain, which accelerates the uncoating of the viral core and thereby interferes with the reverse transcription [24,25]. Among primates, this domain contains four hypervariable regions that have been subjected to a virus-driven diversification and account for the species-dependent retrovirus restriction of TRIM5α [26-28]. The RING and B-box domains of TRIM5α are essential for localizing TRIM5α in specific cytoplasmic 'bodies' and may also be involved in inhibiting the assembly of progeny virions [6,29-31]. The antiviral restriction activity of TRIM19, or promyelocytic leukemia (PML) protein has been demonstrated for retroviruses (HFV, HIV, MLV), but also for an arenavirus (lymphocytic choriomeningitis virus), a rhabdovirus (VSV) and an orthomyxovirus (influenza A) [8,32]. For example, TRIM19/PML binds the HFV Tas protein, a transactivator for HFV transcription, preventing binding of Tas protein to the viral genome and thereby transcription of viral open-reading frames (ORFs) [33]. As expected for proteins involved in antiviral defenses, several TRIM proteins are induced by interferon [34-37], but they can also participate in the induction of interferon synthesis. Thus, TRIM25 is involved in the production of IFN-β through the RIG1-pathway [20].

Here, we characterize a new subset of *trim *genes that were originally discovered in a screen for virus-induced genes expressed by fish [38], and named them *fintrim *for fish novel *Trim*. The *fintrim *genes constitute a unique expansion of *trim *genes in different teleost species, with up to 84 genes identified in zebrafish (*Danio rerio*). In the zebrafish, these genes are located on several chromosomes, with three main clusters on the chromosome 2. The ORFs of this multigene family are highly similar in sequence, but variable in length. The most extended proteins contain a RING finger, two B-boxes, a coiled-coil region and a B30.2 domain. Interestingly, the finTRIM B30.2 domains have evolved under diversifying selection, and are closely related to B30.2 domains present in a NOD-like receptor (NLR) subfamily unique to teleost fish [39,40]. The characterization of the finTRIM subset highlights the evolutionary dynamics of the TRIM family and strongly advocates a role in the innate antiviral response of fish.

## Results

### *finTRIM*, a new group of *TRIM *proteins induced by viruses in rainbow trout

**Figure 1:**
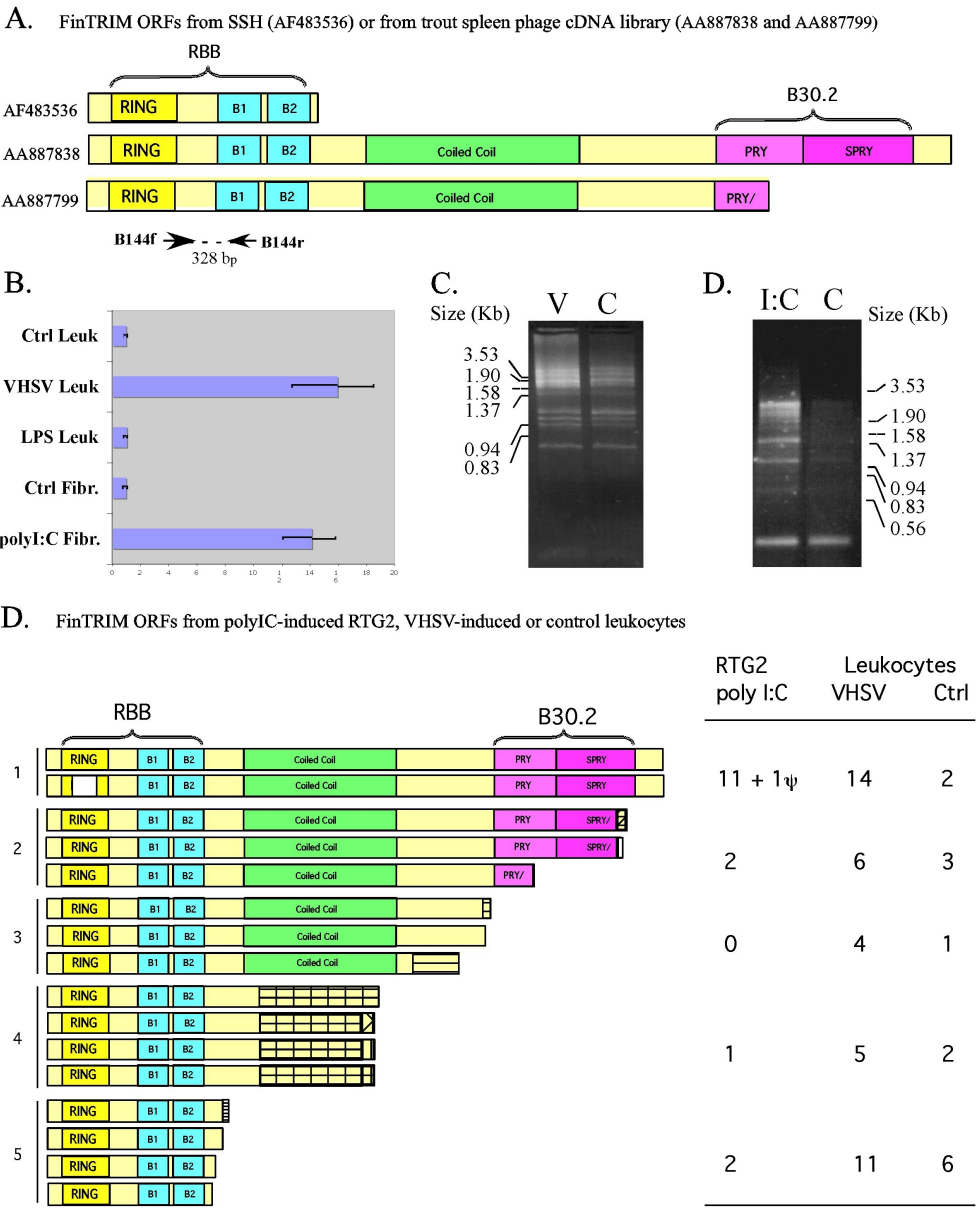
**Variants of rainbow trout finTRIM ORFs**. (A) Schematic representation of the structure of the rainbow trout finTRIMs from SSH and cDNA library. ORFs were predicted from the sequence of full-length transcripts. The RING, B1 and B2 boxes, coiled-coil and PRY/SPRY domains are represented by boxes with specific colors. (B) Quantitative RT-PCR analysis of finTRIM induction in trout leukocytes (Leuk.) incubated with the virus (VHSV) or LPS for 40 h or in RTG-2 fibroblasts (Fibr.) incubated with poly(I:C) for 24 h, respectively. Normalized values for finTRIM expression were determined by dividing the average finTRIM value by the average β-actin value. Then, normalized finTRIM values were subjected to a calibration relative to the basic expression in control (Ctrl) leukocytes or RTG2 cells. The error bars represent standard deviation. (C) Complex profiles produced by 3'RACE amplification of rainbow trout finTRIMs from leukocytes incubated with (V) or without VHSV (Ctrl). (D) 3'RACE profiling of finTRIM from fibroblasts incubated with poly(I:C) (I:C) or without poly(I:C) (Ctrl). (E) Structure of the rainbow trout finTRIM ORFs from the 3'RACE products. Five classes could be distinguished on the basis of motifs and ORF lengths. Striped boxes indicate regions without similarity to the finTRIM consensus, perhaps corresponding to alternative splicing to additional exons. The number of clones corresponding to the different classes from the fibroblasts incubated with poly(I:C), or from leukocytes incubated with or without VHSV are indicated on the right.

In order to identify virus-induced transcripts in fish leukocytes, we previously used the method of subtractive suppressive hybridization on rainbow trout leukocytes that were either incubated with viral hemorrhagic septicemia virus (VHSV) or mock-infected. This approach identified 24 virus-induced sequences [38]. One of them (clone [Genbank:AF483536]) contained a 200 aa ORF with a RING finger and two B-box motifs, resulting in a RBB domain typical of TRIM proteins. No coiled-coil region could be found in this sequence. A naive trout spleen cDNA library was then screened to confirm the structure of this TRIM cDNA and we identified two other full-length sequences (clones [Genbank:AM887799 and AM887838]). These sequences contained a RBB region almost identical to that of the AF483536 clone (96% identity over 200 residues), associated with a coiled-coil region. In addition, the clone AM887799 contained a C-terminal B30.2 domain (Figure 1A). A multiple alignment of these sequences suggested that they did not result from alternative splicing of the same gene. Taken together, these results already suggested that these *trims *belong to a multigenic family with a modular structure. We could not find any obvious counterpart of these *trims *in sequence databases from mammals or other tetrapods. We thus assumed that they may belong to a new subfamily, and named them *fintrims*.

The induction of finTRIM transcripts by the virus was further confirmed using real-time quantitative PCR (Figure 1B) using primers B144f/r located in the RBB region and matching the three clones (and in fact all trout finTRIMs; see Figure 1A for primer location). The induction ratio measured by this real-time PCR therefore corresponded to an average value, and may conceal disparities of the induction level for different genes. An induction ratio higher than 10 was measured after the viral infection in leukocytes or poly(I:C) treatment in fibroblasts, while no induction was noted in leukocytes after incubation with lipopolysaccharide (LPS) from *E. coli*. In LPS-treated leukocytes, IFNγ transcript was induced 7-fold (range 5.8 to 8.4), demonstrating an effective stimulation. These experiments established that at least some finTRIMs are induced by viral infection.

To characterize further the diversity of the finTRIMs, we performed a 3'RACE PCR on VHSV-induced leukocyte cDNA using a universal primer specific for trout finTRIM localized in the highly conserved region in the vicinity of the start codon. These experiments on infected leukocytes revealed a rich profile of amplified bands, which suggested that finTRIM sequences are highly diverse (Figure 1C). A diverse profile was also observed in the absence of infection, but the signal appeared weaker and the profile less complex. To investigate finTRIM expression in a non-lymphoid cell type, similar 3'RACE PCR experiments were performed on the fibroblast cell line RTG2. Poly(I:C) was used to mimic viral infection, since VHSV replicates in these cells and kills them quickly, in contrast to leukocytes. Almost no expression of finTRIMs could be detected in untreated fibroblasts, while a number of bands appeared after poly(I:C) stimulation (Figure 1D). This profile was less diverse than in leukocytes, with a bias towards long transcripts. This observation indicated that different arrays of finTRIMs are expressed in different cells, and corroborated the notion that some forms are inducible by viral infection.

### The rainbow trout *finTRIM *transcripts are highly diverse

To further characterize the finTRIM diversity and to assess the specificity of the 3'RACE PCR amplification, we cloned the PCR products and sequenced clones picked at random. Fifty-four clones from leukocytes and 16 clones from RTG2 cells were completely sequenced. All clones contained a finTRIM sequence, confirming the high specificity of the amplification. The size of finTRIM transcripts was highly diverse, as expected from the 3'RACE profile. Different C-terminal regions of variable length were associated with the N-terminal RBB region shared by all sequences. For some clones, the C-terminus was encoded by short unique sequences unrelated to the longer ones. Based on their length or on the motifs present in the C-terminus, we classified the deduced finTRIM proteins into five different groups (Figure 1E). In fact, although the N-terminal RING/B-box regions of the different finTRIM sequences were highly similar to each other, they were not identical due to single nucleotide substitutions. These substitutions were more frequent than the error frequency due to PCR and sequencing (2 to 5%), and most of them were restricted to a number of conserved sites, which indicates that they did not correspond to artifacts. Therefore, the finTRIM diversity could not be due to alternative splicing of a unique RBB exon to multiple and diverse C-terminal exons. Moreover, the different sequences from leukocyte cDNAs were derived from a homozygous rainbow trout (see Methods), and therefore represent many different genes and not allelic diversity. Taken together, these observations suggest that rainbow trout finTRIMs are encoded by a large number of different genes.

### The *fintrim *genes are highly diverse in zebrafish where they constitute a multigenic family

In order to extend the generality of our findings, we searched for *fintrim *genes in another teleost, the zebrafish *Danio rerio*. Tblastn searches were made on the most recent zebrafish genome assembly (zv7), using as query the trout protein sequence AF483536 containing a RING and two B-box motifs. A set of more than 100 significant hits was obtained. More than half of them, associated with the most significant *E *values, were more similar to the trout *fintrims *than to any other described *trim *gene. Unexpectedly, among low-ranking hits, we also found many genes with sequences most similar to *bloodthirsty (bty)*, a known zebrafish *trim *gene with a B30.2 domain, involved in erythrocyte differentiation and closely related to human *trim39 *[41]. This observation was in accordance with a recent survey of vertebrate TRIM sequences reporting a high diversity of TRIM sequences in the fish genome [17].

As a consequence of our search criteria, all these hits corresponded to N-terminal RBB exons (truncated in three of them). We extended our search by looking for B30.2 domain-encoding exons in the downstream genomic sequence and found one in most cases (>80%). Several genes appeared to be likely pseudogenes, either because of early frameshifts (allelic in some cases, see below), or absence of an identifiable start or stop codon. However, in several instances this may be due to a genome assembly defect or to unusual gene structure with extra undetected exon(s) upstream of the RBB or downstream of the B30.2. The deduced protein sequences were aligned and similarity trees were established (Additional file 1 – Figure S1). The trees obtained with the RBB domains and with the B30.2 domains were highly congruent and allowed us to define two families: one that contained 84 *fintrim *genes (hereafter named *ftr01 *to *ftr84*) and one that contained 33 *bloodthirsty*-related genes (named *btr01 *to *btr33*). Three subgroups, based on apparent phylogenetic age, were defined among the *fintrim *family. The major subgroup (Group A), which includes 65 genes (*ftr01 *to *ftr65*), represents the 'crown group', which appears to have evolved most recently. Group B, including 17 genes (*ftr66 *to *ftr81*) is not monophyletic, and contains genes that appear to have diverged at around the time that the clade that now includes the zebrafish separated from the main teleost lineage. Finally, group C consists of only three genes (*ftr82*, *ftr83*, and *ftr84*), which seem to be the most ancient ones. Within each subgroup, genes were named according to their genomic position. Because the zebrafish genome assembly Zv7 is still imperfect, a few difficulties appeared with the annotation. For instance, the *bty *gene itself was not found in zv7 (*bty *has been mapped to a relatively telomeric position on chromosome 19 [41], and therefore may reside within the same cluster as *btr18*, its closest relative among our dataset). An inverted duplication on chromosome 23 results in the presence of twins (in coding as well as in intronic sequences) for the closely linked *ftr58 *and *ftr59 *genes, which we named *ftr58dupli *and *ftr59dupli*. An assembly gap just downstream of the *ftr20 *gene is probably responsible for its lack of a B30.2-containing exon. Finally, contigs containing four genes (*ftr64*, *ftr65*, *ftr80*, and *ftr81*) are not yet assigned to a given chromosome.

**Figure 2:**
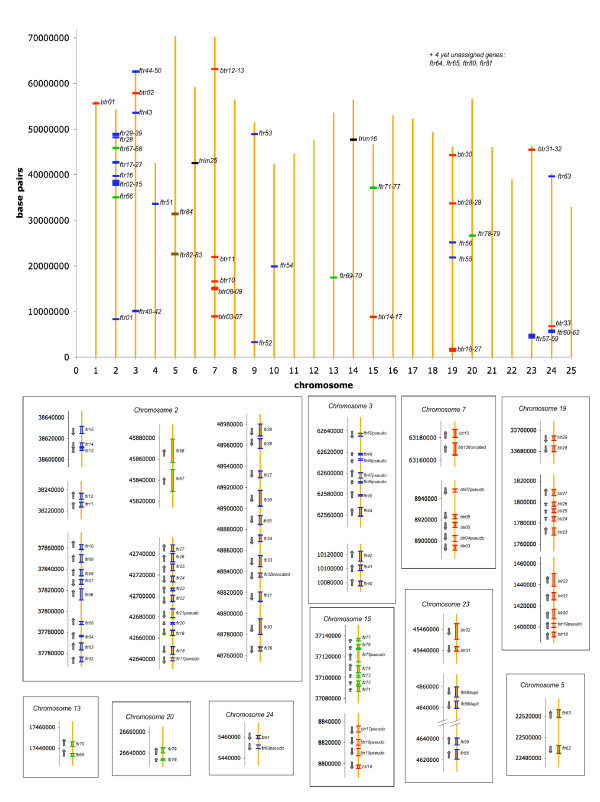
**Genomic location of zebrafish *fintrims *and related genes, based on the zv7 assembly**. Genes are depicted in different colors according to their subgroup: blue for group A *ftrs*, green for group B *ftrs*, brown for group C *ftrs*; red for *btrs*, and black for *trim16 *and *trim25*. Top: global distribution on the whole zebrafish genome; bottom: detailed views of multigenic loci, with gene orientations. Gene limits depicted here correspond to the predicted start and stop codons (see details on Additional file 2 – Table S1) in the zebrafish genome zv7 assembly

The genomic distribution of all *ftr *and *btr *genes is shown in Figure 2: detailed positions are given in Additional file 2 – Table S1. As can be readily observed, most of these genes are arranged in clusters of genes in the same orientation. Half of the *ftr *genes are localized on chromosome 2, with three major clusters. Phylogenetic analysis indicates that genes within a cluster are more related to each other than to genes in other clusters.

In addition to the long and readily detectable exons encoding the N-terminal RBB and C-terminal B30.2 domains, middle exons could be predicted for the majority of genes, with the help of our subsequent RACE analysis (see below) and with GNOMON-predicted sequences deposited in Genbank. For *ftr *genes, organization was very well conserved, with a first RBB-encoding exon of 555 to 594 (coding) bp, two 96 and 234-bp long exons encoding the coiled-coils, two 154 and 60-bp long exons with no clear domain associated, and a final ~545 coding bp exon encoding the B30.2 domain.

**Figure 3:**
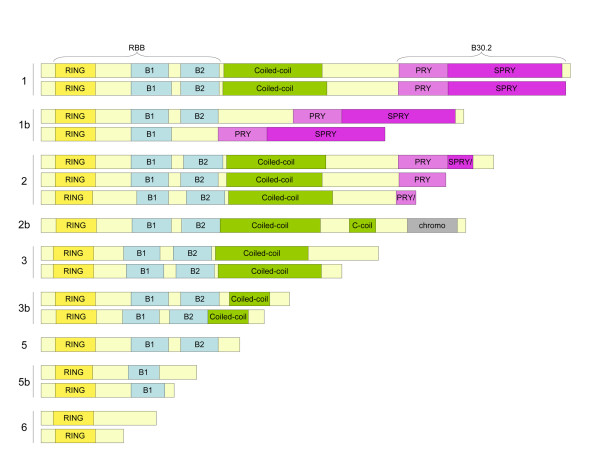
**Variants of zebrafish finTRIM ORFs**. Schematic representation of the zebrafish expressed ftr sequences from group A. Protein sequences were predicted from 3'RACE clones that included a polyA tail. Since the consensus RACE primer was designed in the coding region (in the RING region), the N-termini of the proteins was extrapolated from the corresponding genomic sequences in zv7. Colors of protein domains are as in Figure 1 (plus the grey box representing a chromodomain).

To gain an insight into the expression pattern of zebrafish *fintrim *genes, we designed consensus primers to perform 3'RACE-PCR simultaneously on all members of group A, the largest but also most homogeneous group. RACE-PCR products from zebrafish larvae were cloned and more than 70 clones were sequenced to obtain an approximate image of the relative *fintrim *expression in different settings of virus-induced activation. We tested three different stimuli: IFN1 over-expression, and experimental infection with either Spring Viremia of Carp Virus (SVCV) or a heat-adapted variant of Infectious Hematopoietic Necrosis Virus (IHNV). Results are summarized in Figure 3 and Table 1. Given the very high number of zebrafish *ftr *genes in group A, it would be difficult to get a statistically significant picture of the expression of any single gene, but the aggregated data reveal interesting trends. At the developmental stage examined (72 to 96 hours post fertilization), expression was restricted, or at least highly skewed, towards a subset of genes, most of them on chromosome 2. Genes frequently found in control samples were also expressed in stimulated samples. Some genes, such as *ftr02*, *ftr23 *and *ftr64*, were detected only in stimulated samples. Short and long finTRIM proteins were expressed with or without B30.2 domain, in a striking parallel to what we have found in the trout. Viral or IFN-stimulation did not affect the overall pattern of length distribution, perhaps because RNA was extracted from whole larvae, which may dilute the specific response. Remarkably, alternative splicing did not account for all the diversity in protein size; allelic variation also played a prominent role (note that standard laboratory zebrafish are not inbred, and that pools of larvae were used). For instance, two different groups of *ftr39 *transcripts were found, some coding for canonical, full-length finTRIM proteins, while others, similar in this respect to the allele of the zv7 genome assembly, contain a 2 bp-deletion in the first exon, resulting in a transcript coding for a truncated protein with only a RING domain, irrespective of subsequent splicing.

**Table 1:** ftr expression in zebrafish embryos

**Class**	**Sequences from control embryos (*n *= 32)**	**Sequences from virus or IFN-stimulated embryos (*n *= 35)**
**1**	11 (34%)	9 (26%)
	*ftr14 (2x), ftr15 (3x), ftr39 (3x), ftr56, ftr65 (2x)*	*ftr02 (2x), ftr15, ftr23, ftr39, ftr56 (2x), ftr64, ftr65*
**1b**	-	2 (6%)
		*ftr15, ftr39*
**2**	2 (6%)	3 (9%)
	ftr15*, ftr65*	ftr15* (3x), ftr39
**2b**	1 (3%)	1 (3%)
	*ftr06*	*ftr06*
**3**	5 (16%)	6 (17%)
	ftr14*, ftr15*, ftr19l, ftr51, ftr56	*ftr07, ftr14, ftr19l (2x), ftr39, ftr56*
**3b**	4 (13%)	8 (23%)
	*ftr03 (3x), ftr65*	*ftr02(3x), ftr03 (3x), ftr23, ftr34*
**5**	2 (6%)	-
	*ftr15, ftr65*	
**5b**	7 (22%)	3 (9%)
	ftr15, ftr23, ftr39* (3x), ftr65 (2x)	ftr39*, ftr64*, ftr65
**6**	-	2 (6%)
		ftr56*, ftr64

Group A *finTRIM *genes expressed in zebrafish embryos, as deduced from 3'RACE. Control embryos were either 72 hpf non-manipulated embryos (four sequences); 72 hpf, mcherry-transduced embryos (six sequences); 78 hpf non-manipulated embryos (10 sequences) or 96 hpf non-manipulated embryos (12 sequences). Stimulated embryos were either 72 hpf, zIFN1-transduced embryos (10 sequences); 78 hpf, SVCV-infected embryos (five sequences); or 96 hpf, IHNV-infected embryos (20 sequences). Multiple occurrences of ftr in different classes correspond either to allelic (*) or to splicing variants. Protein isoforms were classified according to Figure 3. Accession numbers [GenBank:AM941305–AM941371].

### The *finTRIM *family is present in many teleost fish but shows various levels of diversity

**Figure 4:**
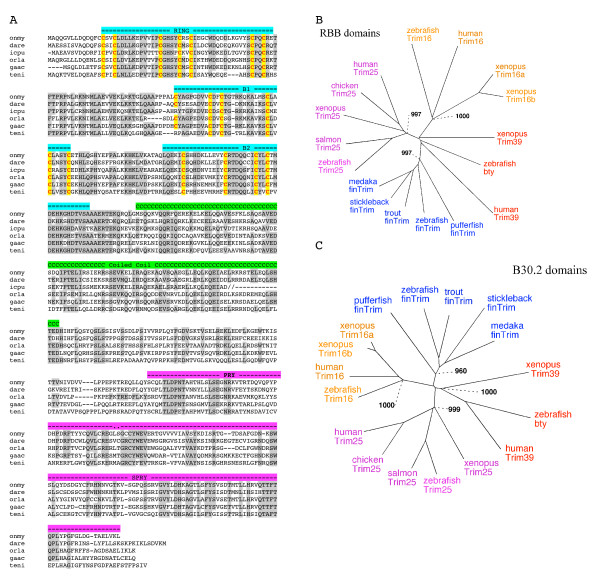
***finTRIMs *represent a teleost-specific multigenic family**. (A). Multiple alignment of representative teleost finTRIM protein sequences. Rainbow trout (onmy) [GenBank:AM887799], zebrafish (dare) (*ftr14*, [GenBank:XM_692536]), channel catfish (icpu) ([GenBank:BM424798], medaka (orla) (Ensembl: ENSORLP00000003320), Stickleback (gaac) (Ensembl, Linkage group III: 14324861–14326501; GENSCAN00000022585), Pufferfish (teni) (Ensembl GSTENT00020235001). (B and C) Phylogenetic trees (NJ, boostrap = 1000) of the finTRIM and their relatives, based on the RBB (B) and B30.2 (C) regions. finTRIM accession numbers are as in (A); other sequences from zebrafish: Bty (bloodthirsty) [GenBank: NP_001018311]; TRIM25, [GenBank: NP_956469]; TRIM16, [GenBank: XR_029737]; from human: TRIM16, [SwissProt: Q99PP9]; TRIM25, [SwissProt: Q14258]; TRIM39, [GenBank: NP_742013]; from chicken: TRIM25, [GenBank: XP_415653]; from salmon: TRIM25 [gene index TC35355 accessible at ]; from xenopus: TRIM16a, [GenBank: AAH74300]; TRIM16b, [GenBank: NP_001086184]; TRIM25, [Ensembl Xenopus genome scaffold255: 821309_819660]; TRIM39, [Ensembl Xenopus genome scaffold709: 241758_272825].

Systematic searches were performed in other available fish genome databases: medaka (*Oryzias latipes*), stickleback (*Gasterosteus aculeatus*) and pufferfish (*Tetraodon nigroviridis*), with tblastn and blastp using the trout and zebrafish conserved N-terminus of finTRIM as bait (Additional file 3 – Table S2). When highly similar hits were found, other finTRIM exons were searched close by, and the predicted sequences were subjected to multiple alignment with trout and zebrafish finTRIMs (Figure 4A). We checked that these sequences retrieved zebrafish finTRIMs or allies when used as a query in the reciprocal blast searches. In medaka, this approach revealed a large number of close *fintrim *homologs, as in zebrafish. The significant hits are mainly clustered on chromosomes 17 (29 hits, including 19 in the same region) and 18 (15 hits). A few genes are also dispersed on other chromosomes (1, 2, 5, 6, 14, 15, 21 and 24). In addition, 24 hits were found which were unanchored to the assembly. Interestingly, the whole ORF is encoded by one exon for most of these hits (47 genes among 55), in contrast to the zebrafish. These intronless genes correspond most probably to retrotransposed genes.

In the pufferfish, the best hit is localized on chromosome 14, and additional homologs are found on chromosomes 7, 9, 17, 18 and 3 (nine hits in total), or among the unanchored sequences.

In the stickleback genome, at least four genes located in the same region of linkage group (LG) III and three genes in LG VII showed high similarity with trout and zebrafish *fintrims*. A *fintrim*-encoding EST ([GenBank:BM424798]) was also identified in the channel catfish. Taken together, these observations suggested that *fintrim *genes constitute a multigenic family in all teleost genomes, with a highly variable number of genes (less than 10 to more than 80 genes).

### The *fintrims *do not possess obvious orthologs in higher vertebrates

We used fish sequences of finTRIM RBB or B30.2 domains with the tblastn program to search for their mammalian homologs in Genbank or genome databases. Both RBB and B30.2 regions from finTRIM sequences and the related sequences found in tetrapods were then subjected to phylogenetic analysis using NJ or parsimony methods, which produced congruent phylogenetic trees. This analysis revealed that the *fintrims *do not possess direct orthologs in mammals or in other tetrapods (Figure 4B and 4C). The mammalian proteins most similar to finTRIMs were TRIM16 and TRIM25. However, reciprocal blast queries, using mammalian TRIM25 sequences, identified in the zebrafish genome one single gene (gb#AY648763) more similar to *trim25 *than to *fintrims*; a relative of this gene was also identified in trout and salmon. Similar queries with mammalian TRIM16 retrieved yet another gene (gb#BC155346). In contrast, no mammalian sequence appeared as a finTRIM ortholog. Orthologs of finTRIMs could not be found in chicken or *Xenopus *either, and therefore seem to be absent from tetrapods.

The phylogenetic analysis showed that finTRIMs sequences from different teleosts grouped in a separate branch supported by high bootstrap values in phylogenetic trees generated by either neighbor-joining (NJ) or parsimony methods for both RBB and B30.2 regions (Figure 4B and 4C). FinTRIMs therefore appear as a teleost-specific subset. In contrast, TRIM16-like and TRIM25-like sequences from fish or clawed frog grouped with their mammalian counterparts, identifying these *trim *genes as fish orthologs of mammalian *trim16 *and *trim25*. Interestingly, in the elephant shark genome, no finTRIM counterpart could be found, while sequences highly similar to TRIM16 and 25 were present (*trim16*: AAVX01048456; *trim25*: AAVX01256120; intermediate *trim16/trim25*: AAVX01115558, available at ). Although this database is still incomplete and shark *trim16/25*-related sequences were partial, these observations reinforce the notion that *trim16 *and *trim25 *are ancient genes while *fintrims *appeared and expanded in the teleost fish.

In addition, since *bty *has been described as the counterpart of *trim39*, we investigated the phylogenetic relationships of the *btr *subgroup and tetrapod *trim39 *with *trim16*, *trim25 *and *fintrims*. Members of the *btr *subfamily (including *bty*) were indeed most similar to tetrapod *trim39*, and reciprocally; they also clustered together in the B30.2-based tree (Figure 4C).

### Conserved syntenies between teleost fish and tetrapods for *fintrim*-related genes

To further investigate the origin of finTRIMs, conserved markers in the vicinity of tetraodon, stickleback or zebrafish *ftr *clusters were identified and used to search for conserved syntenies in other genomes. No clear conserved synteny could be identified between the regions encoding group A or group B finTRIMs in different teleosts, suggesting that *fintrim *genes were subjected to genus or species-specific duplication and expansion episodes during the evolution of teleosts.

**Figure 5:**
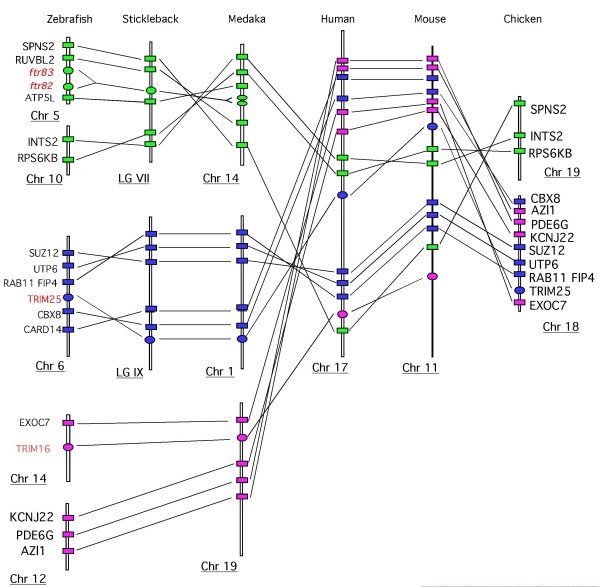
**Conserved synteny of finTRIM-like genes in teleosts**. The locations of *ftr82/83*, *trim25 *and *trim16 *is schematically represented in the context of gene markers in zebrafish, stickleback, medaka, human, mouse and chicken to show conserved synteny groups. The gene locations are indicated (in Kb) in the table, according to the last assembly available for each species at the ensembl website (medaka: HdrR; stickleback: BROAD S1; zebrafish: zv7; human: NCBI 36; mouse: NCBI m37).

In contrast, we could identify a conserved synteny between regions comprising the 'ancient' (group C) *ftr82 *and *ftr83 *genes associated with the markers INTS2, RSPS6, ATP5L, RUVBL2 and SPSN2 in zebrafish, medaka, and stickleback (Figure 5). Conserved syntenies were also observed for markers flanking *trim16 *and *trim25 *in these three species, supporting the hypothesis that these genes are older and kept in a more stable genomic configuration than recent *fintrims*.

Interestingly, the orthologs of all the markers involved in these conserved synteny groups (*ftr82/83, trim25, trim16*) in teleosts were located on the same human chromosome 17, distributed over 70 megabases (Figure 5). In addition, they were also retrieved on mouse chromosome 11 and on the chicken mini chromosomes 18 and 19 that correspond to large regions of the human chromosome 17.

Taken together, these observations in teleosts and tetrapods suggest that the genomic configuration of *trim16 *or *trim25 *(as opposed to *fintrim *genes) cannot be explained by recent sporadic events that occurred in particular species. They rather suggest that the regions containing the ancestral *trim16 *and *trim25 *moved apart in the early fish evolution and were kept as synteny groups on two different chromosomes while most *fintrims *appeared and differentiated by multiple duplications in the fish lineages. The situation is more complex for group C *ftrs 82/83 *(see Discussion).

### The zebrafish *finTRIM *proteins have evolved under positive selection

**Figure 6:**
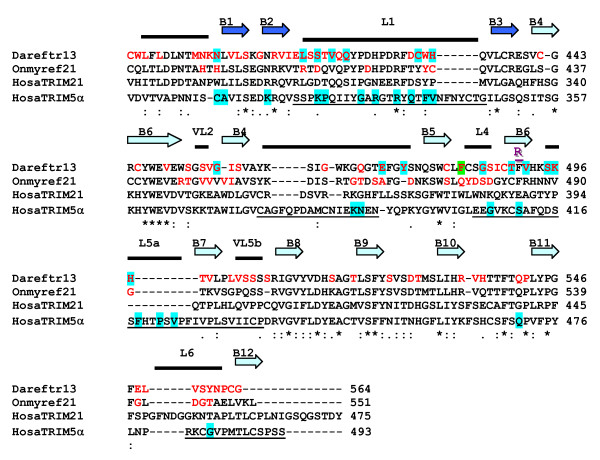
**Positive selection in the B30.2 domain**. Distribution of hypervariable and positively selected residues in a multiple alignment of B30.2 domains from representative zebrafish and rainbow trout finTRIMs (Dareftr13: [GenBank: XM_695031], Onmyref21: [GenBank: XM_695031], human TRIM21 (HosaTRIM21) and TRIM5α (HosaTRIM5α). Sites that are less than 80% conserved among zebrafish or among trout sequences are indicated in red. Positively selected sites (among zebrafish finTRIMs: this study; among primate TRIM5α : previous work of Sawyer et al [44]) are boxed in blue when detected under models 2a and 8. One site was positive under M8 but not under M2 and is boxed in green. β-strands identified from the TRIM21 structure B30.2 sequence are indicated by dark (PRY) or light (SPRY) blue arrows [16]. The variable loop-connecting strands are named VL1–VL6. The four hypervariable regions of the TRIM5α B30.2 domain are underlined [27]. The detailed PAML results for each position under positive selection are available in Additional file 6, Table S4. The purple 'R' indicates the recombination site identified by GARD.

To gain further insight into the meaning of finTRIM sequence variability, we analyzed the pattern of variable positions, in the context of the tertiary structure of the B30.2 domain that has been determined for human TRIM21 [16,42]. The B30.2 domain forms a distorted β-sandwich of two antiparallel β-sheets, made up by the PRY and SPRY subdomains [16,42,43]. The β-strands are connected by six variable loops that define regions of hypervariability and form the ligand-binding surface in TRIM5α and TRIM21. We determined variability in finTRIM B30.2 by performing two multiple alignments of both trout finTRIM B30.2 and zebrafish finTRIM group A B30.2 sequences (see Additional file 4 – Figure S2) and determined site-by-site variation. We then aligned the B30.2 domains of trout and zebrafish finTRIM with human TRIM5α and TRIM21 (Figure 6). The trout finTRIM B30.2 sequences are rather conserved and the majority of variable sites, 23 of 29, are located in the regions that correspond with the variable loops of TRIM21. Fifteen variable sites of trout correspond with regions that are hypervariable in primate TRIM5α. The zebrafish finTRIM B30.2 sequences are even less conserved with 78 variable sites that are also associated with the predicted variable loops of TRIM21 or with the hypervariable regions of TRIM5α, albeit more loosely.

For primate TRIM5α it has been demonstrated that the B30.2 domain has evolved under diversifying selection pressure, with the positively selected sites predominantly located in the four regions that are hypervariable among primate TRIM5α [27,44]. We investigated whether the zebrafish *fintrim *genes have also evolved under diversifying selection. We used a test that is based on the estimation of synonymous (*dS*, silent mutations) and non-synonymous (*dN*, amino acid-altering) substitution rates of all codons among a set of sequences. The ratio ω = *dN*/*dS *is an indication for negative selection (ω < 1), neutral evolution (ω = 1), or positive selection (ω >1). If an amino acid change is neutral, it will be fixed at the same rate as a synonymous mutation, and ω = 1. If the amino acid change is deleterious, purifying (negative) selection will reduce its fixation rate, thus ω < 1. An amino acid change is fixed at a higher rate than a synonymous mutation (ω >1) only when it offers a selective advantage. The site-specific model within the Phylogeny Analysis by Maximum Likelihood (PAML) software package allows heterogeneity in evolutionary pressure along a protein encoding sequence and can identify the specific sites that are under positive selection. Two models of substitution distribution, M2a and M8, can be used to test the positive selection hypothesis against the nested null models: M2a against M1a and M8 against M7.

We took the complete sequences of zebrafish finTRIM group A B30.2 domains and analyzed them with the PAML models M1a, M2a, M7 and M8. A value of ω >1 was detected for 15.6% of sites under M2a and 17.6% of sites under M8 (see Table 2). The likelihood-ratio test (LRT) was significant with *p *< 0.001 for both models (see Table 3). The estimation of substitution rates by PAML is based on branch lengths of sequences in the phylogenetic tree. As a result of recombination, sites within one sequence are no longer similar in branch length and this can interfere with the results of PAML since this model assumes that all sites within a sequence are similar in branch length. To investigate whether the detection of positive selection was not perturbed by recombination, we implemented the algorithm PARRIS on our dataset. With the PARRIS program, a partitioning approach is used and site-to-site variation in both synonymous and non-synonymous rates is integrated in the M1a and M2a models. We analyzed the zebrafish finTRIM group A B30.2 sequences with PARRIS and could still detect positive selection by the LTR with *p *< 0.001, indicating that whether or not recombination did occur, the B30.2 has evolved under positive selection. To search for recombination sites, we employed the program GARD. The algorithm subdivides the sequence alignment in putative non-recombinant fragments, phylogenies are inferred for each fragment and the goodness of fit is assessed by Akaike's information criterium (c-AIC), predicting whether or not the fragments are derived from two different ancestor sequences due to recombination. We searched for either two or up to 20 breakage points and could identify one breakage point by both searches, with Δc-AIC = 42.96 under the two-breakage-point model and Δc-AIC = 28.67 under the 20-breakage-point model (see Additional file 5 – Table S3).

**Table 2:** PAML results

Region^1^	*n*^2^	*c*^3^	Parameters in ω distribution under M2a^4^		Parameters in ω distribution under M8^5^	
B30.2	38	145	ω_>1 _= 2.98787	***p***_>1_** = 0.15644**	ω_>1 _= 2.64795	***p***_1_** = 0.17627**
complete 1–435			ω_1 _= 1.000	***p***_1 _= 0.25983		***p***_0 _= 0.82373
			ω_<1 _= 0.18514	***p***_<1 _= 0.58373	***p ***= 0.64985	*q *= 1.07859
						
B30.2	38	95	ω_>1 _= 2.79395	***p***_>1 _= 0.15969	ω_>1 _= 2.38329	***p***_1 _= 0.20431
fragment 1–285			ω_1 _= 1.000	***p***_1 _= 0.28631		***p***_0 _= 0.79569
			ω_<1 _= 0.17036	***p***_<1 _= 0.554	***p ***= 0.62590	*q *= 1.00411
						
B30.2	38	50	ω_>1 _= 3.28071	***p***_>1 _= 0.09143	ω_>1 _= 2.84563	***p***_1 _= 0.09925
fragment 286–435			ω_1 _= 1.000	***p***_1 _= 0.28492		***p***_0 _= 0.90075
			ω_<1 _= 0.17611	*p*_<1 _= 0.62365	*p *= 0.70827	*q *= 1.26680
						
RBB	55	155	ω_>1 _= 2.1211	***p***_>1 _= 0.06286	ω_>1 _= 1.58831	***p***_1 _= 0.07109
complete 1–465			ω_1 _= 1.000	***p***_1 _= 0.44597		***p***_0 _= 0.92891
			ω_<1 _= 0.11812	***p***_<1 _= 0.49117	***p ***= 0.47665	*q *= 0.86554
						
RBB	55	78	ω_>1 _= 2.53603	***p***_>1 _= 0.06002	ω_>1 _= 1.98626	***p***_1 _= 0.06121
fragment			ω_1 _= 1.000	***p***_1 _= 0.43598		***p***_0 _= 0.93879
1 – 234			ω_<1 _= 011843	***p***_<1 _= 0.50400	***p ***= 0.93879	*q *= 1.04448
						
RBB	55	76	ω_>1 _= 2.05674	***p***_>1 _= 0.02961	ω_>1 _= 1.49995	***p***_1 _= 0.02779
fragment 238–465			ω_1 _= 1.000	***p***_1 _= 0.46960		***p***_0 _= 0.97221
			ω_<1 _= 0.12722	***p***_<1 _= 0.50079	***p ***= 0.47756	*q *= 0.81657

^1 ^For sequence fragments, the numbers correspond with the position of first and last nucleotides in the alignment with excluded gaps.

^2 ^*n*, the number of sequences in the alignment and tree.

^3 ^*c*, the number of codons.

^4 ^Parameters determined under M2a with ω the ratio of non-synonymous rates (*dN*) and synonymous rates (*dS*) and *p *the corresponding proportion of sites for each ω-class.

^5 ^Parameters determined under M8 with ω the ratio *dN*/*dS*, the corresponding proportion (*p*_1 _= 1-*p*_0_) of sites and *p*- and *q*-estimates in the β (*p*, *q*)-distribution.

**Table 3:** Results of LRT for positive selection

Region^1^	Model^2^	2 Δ lnL	*p*-value	No sites
B30.2 complete	PAML M1a-M2a	208.21	*p *< 0.001	16
	PAML M7–M8	231.62	*p *< 0.001	21
				
B30.2 complete	PARRIS M1a-M2a	29.38	*p *< 0.001	ND
				
B30.2 1–285	PAML M1a-M2a	124.15	*p *< 0.001	13
	PAML M7–M8	129.55	*p *< 0.001	14
				
B30.2 286–485	PAML M1a-M2a	41.33	*p *< 0.001	3
	PAML M7–M8	47.95	*p *< 0.001	3
				
RBB complete	PAML M1a-M2a	25.25	*p *< 0.001	4
	PAML M7–M8	18.20	*p *< 0.001	2
				
RBB complete	PARRIS M1a-M2a	20.95	*p *< 0.001	ND
				
RBB 1–234	PAML M1a-M2a	21.04	*p *< 0.001	3
	PAML M7–M8	17.94	p < 0.001	3
				
RBB 238–465	PAML M1a-M2a	3.73	*p *= 0.155	0
	PAML M7–M8	1.57	*p *= 0.455	0

^1 ^For fragmented regions, numbers correspond with positions of first and last nucleotides in the alignment.

^2 ^The models M1a, M2a, M7 and M8 were employed, using either the program PAML or program PARRIS.

We re-analyzed our zebrafish finTRIM group A dataset of B30.2 by subdividing the alignment in two parts, containing the sequence regions before or after the detected recombination point. For both regions, we detected positive selection, with *p *< 0.001 in the LRT under M1a-M2a and M7–M8 models (see Table 2 and Table 3). The specific sites under positive selection according to the models 2a and 8 were identified by a Bayesian approach. For the B30.2 domain we were able to identify 16 sites under both model 2a and 17 under model 8. Fourteen sites were located in regions corresponding to the predicted variable loops of TRIM21. In addition, the majority of the sites were located within the regions corresponding with the four hypervariable regions described for TRIM5α, with six sites falling in the hypervariable region 1, one in region 2 and six in region 3. (See Figure 6 for positions of sites and Additional file 6 – Table S4 for posterior probabilities).

We used a similar approach to detect positive selective sites in the RING and two B-box domains. First we analyzed with PAML the complete dataset from all RING-B-box sequences from zebrafish finTRIM group A. Positive selection was detected for 6.3% of sites under M2a and 7.1% of sites under M8 with *p *< 0.001 in the LTR of both M1a-M2a and M7–M8. With PARRIS we confirmed that the RBB has evolved under positive selection with *p *< 0.001 in the LTR of M1a-M2a. We used GARD to search for recombination and we also identified a breakage point, with a significant value of Δc-AIC = 254.63 under the two-breakage-point model and Δc-AIC = 263.22 under the 20-breakage-point model. We therefore divided the RBB multiple alignment into two segments and re-analyzed the sequences located before and after the breakage site using PAML. For the sequences located before the predicted breakage point we found three sites under positive selection under M2a and under M8, with *p *< 0.001 in the LTR under both nested models. These sites are located just upstream of the RING motif. For the region after the breakage point, the test for positive selection was no longer significant, with *p *= 0.155 in the LTR of M1a-M2a and *p *= 0.455 in the LRT of M7–M8, (see Table 2).

Taken together, these results firmly establish that the loops of the zebrafish Group A finTRIM B30.2 domains have been diversified under positive selection, as previously described for the sites determining virus specificity in TRIM5α, suggesting a selective pressure on this domain for binding to diverse ligands. A few positions located close to the RING motif are also subjected to diversification.

### Exon shuffling of B30.2 domain occurred between *finTRIMs *and NLR

**Figure 7:**
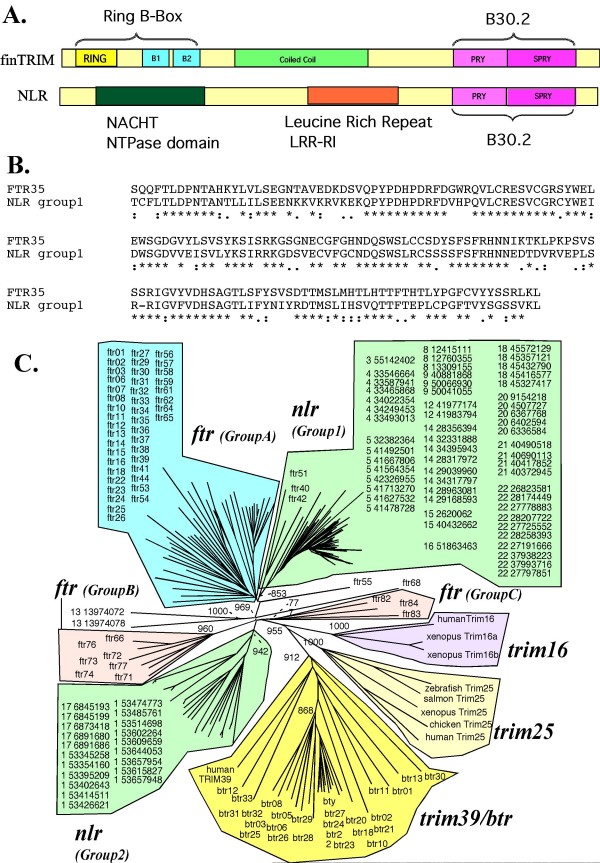
**Distance tree of finTRIM B30.2 domains and related sequences**. The domain organization of finTRIM and NLR proteins is represented in (A). A typical alignment of the B30.2 protein sequences from group A ftr (ftr35) and NLR (chr5_32382364) is shown in (B). B30.2 domains of the zebrafish *fintrim*s and *btrs *as well as those extracted from related *trim*s of other vertebrates (human and xenopus *trim*16, human *trim*39, Atlantic salmon, zebrafish, xenopus, chicken and human *trim*25; see Figure 4 for accession numbers) were aligned with related B30.2 sequences from *nlr*s, and a distance tree was produced using mega4 (NJ, boostrap = 1000); high bootstrap values of key nodes are indicated (C).

Since finTRIM B30.2 features suggest that they may be involved in interactions with diverse ligands, similar B30.2 domains may be used in other TRIM subfamilies for comparable purposes. We therefore searched the zebrafish genome for sequences closely related to typical finTRIM B30.2 domains. Unexpectedly, the closest counterparts of finTRIM B30.2 sequences were not found in other TRIM, but were associated with NACHT and leucine-rich repeats (LRRs)-ribonuclease inhibitor (RI)-like motifs in NLR proteins (Figure 7A). These proteins, which possess a B30.2 and a NACHT domain and are very likely involved in innate immunity, have been identified very recently; such a combination is not found in mammals [40]. While the protein sequences of B30.2 from Group A ftrs were about 40% similar to B30.2 domains from close TRIM relatives such as bloodthirsty or TRIM25, they were 55 to 65% similar to B30.2 from these NLR proteins, and about 50 to 55% similar to those of the Group B or C ftr sequences (Figure 7B). A phylogenetic analysis showed that B30.2 sequences of group A finTRIM and a subgroup of NLR (named here group 1) are joined as a cluster supported by a high bootstrap value (Figure 7C). This cluster is quite distinct from that of other TRIMs, including group B or group C *ftr *and *btr*, or even from the rest of B30.2-bearing NLRs (group 2), suggesting an exon shuffling event between group A *fintrims *and group1 *NLRs*.

## Discussion

We have described here a large set of closely related genes and transcripts that contain the three motifs typical of TRIM proteins, namely the RING zinc finger, two B-boxes and a predicted coiled-coil region. Their close relatedness allowed us to group these sequences in one multigene family, the finTRIMs. The *fintrim *genes were identified in all teleost fish species for which a genome database is available. In addition, we characterized a number of transcripts in rainbow trout and zebrafish, confirming that these genes are actively transcribed. Although the gene numbers vary among the different teleost species, the wide distribution of the *fintrim *genes suggests an important role, one in which diversity offers a selective advantage to the host. Since finTRIMs are specifically induced by viruses and poly(I:C), they probably play a role in antiviral immunity, as several other TRIMs do in mammals.

### *FinTRIM *diversity suggests that they recognize multiple ligands

The highly diverse finTRIMs are encoded by a large number of genes. This TRIM subset is a teleost-specific group, well distinct from other TRIMs shared by fish and mammals as TRIM16, TRIM25, TRIM39 and others. Next to the variety generated by the large number of genes, we demonstrate here that the B30.2 domain of *fintrim *genes has evolved under diversifying selective forces. This indicates that these proteins bind a diverse range of ligands, making an immune function very plausible. Consistent with a role in immune recognition, our current data suggest that there is a high allelic polymorphism among zebrafish *fintrim *genes. There are numerous examples of large polymorphic multigene families that are involved in innate immunity. The proteins they encode either recognize a variety of microbial patterns, or bind diverse receptors of the host immune system and participate in the tuning of complex activation pathways. Such receptors include some mammalian TRIMs [45-47], but also the mammalian killer cell immunoglobulin-like receptors (KIR) [48] and Ly-49 related proteins [49], chicken Ig-like receptor (CHIR) [50,51], fish novel immune-type receptors (NITR) [52,53], fish leukocyte immune-type receptors (LITR) [54], and sea urchin toll-like receptors [55].

If finTRIMs are specifically involved in virus recognition or act as virus-restricting molecules, the high number of *fintrim *genes opens the possibility for parallel and simultaneous selection by different viruses [46].

### Signatures of positively selected residues in *finTRIM *B30.2 equate with canonical motifs of the virus-binding sites in TRIM5α

The finTRIM B30.2 domain contributes dominantly to finTRIM diversity, and seems to be generally similar in structure to the B30.2 domain of TRIM21. Among the sequences of the multiple finTRIMs described in both trout and zebrafish, the variable sites are predominantly located in the variable loops of the domain, in a way that strongly suggests that the B30.2 domains interacts with their ligands as TRIM5α does for viral proteins. In particular, the sites identified in zebrafish as subjected to significant diversifying ('positive') selection were concentrated in the variable loop 1, between the β-strands 2 and 3. This loop was earlier designated as a 'hotspot' region, since sites within this region determine the lentivirus restriction-specificity of TRIM5α and mutations in this region are correlated with the disease susceptibility associated with TRIM20 (familial Mediterranean fever) and TRIM21 (an autoantigen in multiple diseases) [16]. Such a distribution of sites diversified under positive selection in the loop 1 (loop β2–3) of the B30.2 domain strongly supports both the reality of a diversifying selection and the diversity of *ftr *B30.2 ligands.

In addition, we also found several sites under diversifying selection within the RBB domain, suggesting that additional sites may also be involved in binding of a(nother) ligand. A few sites were earlier shown to have evolved under diversifying selection in the coiled-coil region of proteins TRIM5α and TRIM22 [44], but positive selection of RING and B-box domains was not reported. Besides, the roles of the RING and B-box domains in TRIM function are still not fully understood, although deletion or mutations can abrogate the activity of TRIM5α, and the significance of the positively selected RBB residues in *ftrs *remains therefore elusive. In this context, one may also question the function of the *finTRIM *genes that do not contain a B30.2 domain (one out of five in the zebrafish, but probably more in trout). It is tempting to consider them as potential inhibitors/regulators of an antiviral response triggered by B30.2-containing genes.

### The evolutionary affinities of *finTRIM *B30.2 domains suggest a domain shuffling between NLR and TRIM molecules

**Figure 8:**
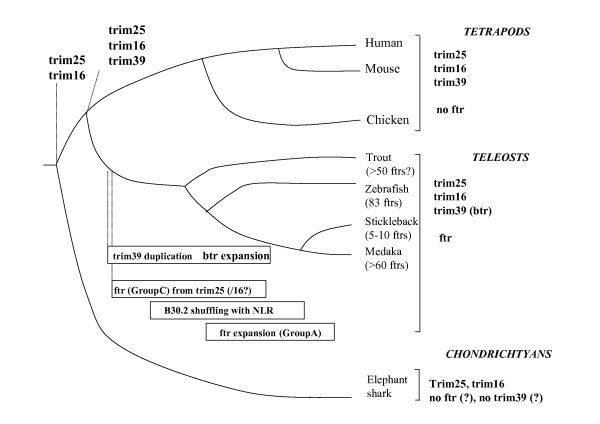
**Tentative evolutionary pathway of *fintrims *and their relatives in vertebrates**. The genes identified in the genomes of different vertebrate groups are indicated on the right. Since the current draft of the elephant shark genome is still partial, the absence of *fintrim *and *btr *is unsure, which is indicated by '?'.

An interesting finding was the close relationship of finTRIM B30.2 domains with the B30.2 domains present in a subset of NLR proteins recently described in the zebrafish. NLR proteins, also known as CATERPILLER, NACHT or NOD-LRR proteins, are large cytoplasmic proteins involved in inflammation and apoptosis. They are characterized by a NACHT domain and a leucine-rich repeat (LRR) region at the C-terminus and vary in their N-terminal effector domain, which is a CARD, pyrin or TIR domain. As for TRIM proteins, the physiological functions of NLRs are diverse, and several NLRs are inhibitors/activators of the inflammatory and immune responses. For example, NALP3/cryopyrin is a key component of the inflammasome and inhibits TNF- and TRAF6- induced NFκB activation [56], while NOD2 plays a role in NFκB activation [57]. Large groups of novel, fish-specific NLR proteins have been recently described in fish and appear to be highly related within each species, indicating recent species-specific expansions [39]. Some of these teleost NLR proteins contain the NACHT and LRR domains at the C-terminus in combination with a B30.2 domains, as the NLR-C described in [40]. The close relatedness of these B30.2 domains to the group A finTRIM B30.2 domain suggests that the corresponding exon(s) have been subjected to shuffling between NLR and group A finTRIM, that is, during *ftr *evolution (Figure 8). The exchange of a ligand-recognition module evolving under positive selection with another protein family mainly involved in inflammation, immune regulation and possibly pathogen sensing constitutes another argument supporting an immune function for group A finTRIMs.

### Phylogenetic analyses suggest that *ftr *appeared and diversified during the teleost evolution while *trim16/25/39 *are more ancient genes common to all vertebrates

The *fintrims *and their relatives (*trim25*, *trim16*, *trim39*) followed different evolutionary pathways (Figure 8). Both teleosts and mammals possess single orthologous *trim25 *and *trim16*, as evidenced by phylogenetic analysis and conserved synteny. Sequences coding for partial *trim16 *and *trim25 *were also identified in the elephant shark genome, confirming that these genes were already present in the early vertebrates. In contrast, *fintrims *seem to be unique to teleost fish and could not be found in any other group of animals. Since they were present in all fish for which a significant amount of genomic data was available, *fintrims *most probably appeared during the early evolution of teleosts. While they are represented by large gene sets in zebrafish, salmonids and medaka, several fish species such as fugu or stickleback only possess a few copies of *fintrim *genes. Thus, *fintrim *genes have been probably subjected to parallel – and independent – duplication events in the different branches of teleosts. This hypothesis is strengthened by the fact that zebrafish *ftr *genes were generally most similar to their immediate neighbors, suggesting tandem duplication within clusters rather than *en bloc *duplication as the main gene amplification mechanism.

This view is supported by the absence of obvious synteny between the regions encoding almost all finTRIMs in different teleosts. Also, diverse mechanisms of gene spreading appear to have been used in different species, since many medaka *fintrims *are intronless in contrast to zebrafish *ftr*, suggesting that one or more retro-transposition events have been involved in the multiplication of these sequences. This observation is a good argument for a fast expansion in this species, and reinforces the idea of strong selection pressures towards finTRIM diversification. Such selection pressures exerted by species- or family-specific viruses are expected to be highly variable between different fish taxa. Whether another TRIM set constrains or balances the evolution of finTRIMs, as described for anti-retroviral TRIM5α and TRIM22 [44], remains to be established. Also, the B30.2 exon shuffling has complicated the *fintrim *evolutionary pathway.

finTRIM Group C is closest to other TRIMs in the phylogenetic trees, suggesting that they appeared during the early evolution of teleosts. They are present in the different fish investigated so far but no counterpart was found in other vertebrates. Interestingly, a conserved synteny of *ftr82/83 *and a few neighboring markers was established in zebrafish, stickleback and medaka, indicating that they were kept in a more stable genomic context than other *fintrims*; it is also worth noting that in the medaka, these genes have retained the six-exon structure. Also, several markers located close to *ftr82/83 *in fish possess counterparts on the human chromosome 17, where both *trim16 *and *trim25 *are located together with the markers defining their own conserved syntenies among vertebrates. This loose association is unlikely to have occurred by chance, and may suggest that certain group C *fintrims *could constitute an intermediate between the main *fintrim *family and the older *trim *genes (presumably *trim25*; *trim16 *is an unlikely candidate since its RBB domain is truncated) from which they appeared by duplication. This hypothesis fits well with the whole-genome duplication that occurred in the beginning of teleost evolution [58]. Along the same line, *ftr82 *and *ftr83*, together with *ftr84*, are the most similar to *trim25 *among the *fintrim *relatives (see *ftr/btr *tree in Additional file 1 – Figure S1, and Figure 7). TRIM25 is involved in IFN signaling, but interacts with endogenous RIG-I and not with viral proteins [20]. It is therefore tempting to speculate that *ftr82 *and *ftr83 *have been restrained from duplications and diversifying selection by such a functional specialization. Such a contrasted evolutionary history has been observed for example for the CytP450 superfamily: enzymes with endogenous substrates are phylogenetically stable, while xenobiotic detoxifiers are encoded in unstable gene islands that appeared by tandem duplication [59]. It could be argued that the quick radiation of group A *fintrims *was triggered when a *trim25*-like *ftr *acquired a B30.2 exon maybe derived from a NLR gene, allowing the newly created finTRIM to directly detect a pathogen motif. Such an event would have occurred after the teleost-tetrapod split, and perhaps even after the divergence of main teleost lineages, as illustrated in Figure 8. It has to be underlined here that the phylogenetic analysis does not support a monophyletic origin of group B *fintrims*, which therefore probably constitute the tracks of several duplication/differentiation events in the finTRIM group. Such duplication events have been suggested to explain the expansion of TRIM genes in fish and other species [17]. A recent extensive survey of TRIMs divided these proteins into two large groups: an evolutionary conserved group I comprising TRIM with various C-terminal domains, and a more recent group II that groups sequences containing a B30.2 domain and showing species-specific diversification. The presence of two B-box motifs – although the B-box-1 is rather degenerated – and the sequence similarity to TRIM16 and TRIM25 suggest that finTRIMs may be closer to group I. However, the finTRIM evolutionary pathway described here fits better the properties of the group II. Our observations seem to reflect a fish-specific evolutionary pathway of a TRIM subset derived from ancestral group I members by an ancient duplication.

The evolutionary pathways of *trim39 *is rather different since it was retrieved as a single gene in mammals but as a multigene set in several teleosts. TRIM39 is a member of the group II as described in [17] and the diversification observed in the zebrafish is well in accordance with the evolutionary properties of this group. Thus, there are more than 30 orthologs of *trim39 *in zebrafish. We named these genes *btrs *for 'bloodthirsty-like TRIMs', as one of them is known as *bloodthirsty*, a gene involved in erythropoiesis [41]. These observations indicate that *trim39 *was already present in the common ancestor to fish and mammals, but was subjected to a successful expansion by duplication in at least some lineages of teleosts.

## Conclusion

In conclusion, our results indicate that the finTRIM family has been subjected to a quick, extensive diversification by duplication and specialization under positive selection exerted on positions concentrated in the B30.2 domain. The sharing of B30.2 domains with NLR emphasizes the shuffling of a putative target-binding module between two major protein families involved in immune recognition of pathogen specific motifs [39,40]. While the targets of finTRIMs have yet to be identified, this first survey suggests that finTRIMs are involved the antiviral innate immune system. Our future work will be aimed at a further understanding of the biological functions of the finTRIMs.

## Methods

### Trout leukocyte preparation

Rainbow trout (*Oncorhynchus mykiss*) were raised in the Jouy-en-Josas experimental fish facility. The fish were sacrificed by overexposure to 1‰ 2-phenoxyethanol (2-PE). The entire pronephros were removed aseptically and dissected. Cells from the pronephros of a single fish were deposited on a Ficoll solution (Lymphocyte separation medium [*d *1.077]; Eurobio, Les Ulis, France) and centrifuged 10 min at 900 *G*. The leukocyte fraction at the Ficoll-medium interface was collected and subsequently used in the finTRIM stimulation experiments and mRNA isolation. Homozygous rainbow trout clones were produced using a gynogenesis-based strategy [60]. A population of doubled haploids was first established, using a mitotic gynogenesis procedure as described. At the next generation, homozygous clones (G-clones) were obtained using meio-gynogenetic reproduction of individual homozygous doubled haploid females. Within a G-clone, some progeny were sex-reversed by early hormonal treatment to obtain functional XX males, and these animals were crossed with females from the same G-clone to produce N-clones. Such animals (N-clones) were used in this study.

### Cells and viruses

Trout RTG-2 and carp EPC cell lines were used for virus production and titration. They were cultured in BHK-21 medium (Invitrogen-Gibco, Leek, The Netherlands), supplemented with 10% (V/V) fetal calf serum (FCS) (Eurobio), 10% (V/V) tryptose broth, streptomycin (50 μg/ml) and penicillin (50 units/ml). African green monkey COS-7 cells were used for rainbow trout recombinant interferon production. They were cultured in Dulbecco's Modified Eagle's Medium (Eurobio), supplemented with 10% (V/V) fetal calf serum. COS-7 cells were transfected with an expression plasmid encoding trout interferon (from AY788890) using FuGENE 6 Transfection Reagent (Roche, Neuilly, France) and supernatant was collected and titrated. Interferon at 1000 U/ml was used to study induction kinetics. Cycloheximide (100 μg/ml) (Sigma Aldrich, Saint-Quentin, France) was used to block cell protein synthesis. Viral hemorrhagic septicemia virus (VHSV) strain 07–71 [61] was inactivated by overnight treatment with diluted (1/4000) β-propiolactone (BPL). For the stimulation of finTRIM expression, 50 μg/ml Poly(I:C) (Sigma-Aldrich), 100 μg/ml of *Eschericha coli *lipopolysaccharide (LPS from *E. coli *0127B8; Sigma-Aldrich) were used. Spring viremia of carp virus (SVCV) and a heat-adapted variant of infectious hematopoietic necrosis virus (IHNV) were used for zebrafish infection experiments. SVCV was diluted to 10^7 ^pfu/ml and IHNV to 5×10^6 ^pfu/ml in PBS containing 0.1% phenol red; viral suspensions were kept as much as possible on ice.

### Zebrafish embryos experiments

Zebrafish (*Danio rerio*) of the AB strain, initially obtained from the Zebrafish International Resource Center (ZIRC, Eugene, OR) or F1 derived from this stock were raised in the Institut Pasteur facility and mated to obtain eggs. IFN-over-expressing embryos were obtained as described in [62]. Embryos were injected at the one-cell stage with 12 pg of plasmid DNA driving expression of zebrafish IFN1; as a control for successful injection, the DNA solution also included a plasmid driving expression of the fluorescent mCherry protein. As controls, some embryos were injected with the mCherry plasmid alone. Embryos were then allowed to develop at 28°C. At 24 hpf (hours post-fertilization) they were sorted under a fluorescence stereomicroscope to retain only mCherry-expressing animals; abnormally developing embryos were also discarded. At 72 hpf, the larvae were euthanized with 2-PE and homogenized in Trizol reagent (Invitrogen) for RNA extraction. For viral infections, non-manipulated larvae that had been left to develop for 3 days at 24°C were dechorionated, anesthetized with Tricaine (Sigma-aldrich), and microinjected with 1 nl of viral suspension in the venous plexus located just posterior to the cloaca. They were then incubated at 24°C for 24 h in the case of SVCV, or 48 h in the case of IHNV (yielding larvae at a developmental stage corresponding roughly to 78 hpf and 96 hpf at 28.5°C, respectively); at this time point infection was well advanced, but all embryos were still alive. Embryos were then sacrificed and treated with Trizol as above. Uninjected control larvae, incubated at the same temperature and for the same durations, were processed in parallel from the same clutches. Ten to fifteen larvae were included for each point.

### RNA isolation, cDNA synthesis, 5' and 3'-RACE PCR

Total RNA was extracted with the Trizol reagent according to the manufacturer's instructions, then treated with 5 units of RNAse-free DNase (Invitrogen) to remove any remaining genomic DNA. cDNA was synthesized from total RNA using either an oligo(dT) primer or the SMART PCR cDNA Synthesis Kit (Clontech BD, Saint-Germain-en-Laye, France). The 5'-rapid amplification of cDNA ends 5'-RACE PCR and 3'-RACE PCR were performed using the SMART RACE cDNA Amplification Kit (Clontech BD), according to the instructions of the manufacturer. 5' and 3'-RACE PCRs were performed with relevant specific primers (see Table 4) and the universal primers from Clontech. In rainbow trout, the 3'-RACE PCR was performed from VHSV-induced leukocyte cDNA using a universal primer specific for trout *finTRIM *localized in the highly conserved region around the start codon. This conserved region was confirmed through EST analysis and sequencing of 10 clones generated by a 5'-RACE experiment using the same template. For zebrafish samples, 3'-RACE was performed using Invitrogen's GeneRacer kit, with two rounds of amplification (round one with primers zftruniv1S and GeneRacer 3' primer, round two with zftruniv2S and GeneRacer nested 3' primer); the consensus primers are located in the RING domain.

**Table 4:** Primers

**Rainbow trout**	Primer sequences
***Q PCR***	
OmOLIB144F	AGGACATGAGGGCTTTCTGCTT
OmOLIB144R	GGACCAGGACCAGTTCTGTTGT
IFNGF	GCTGTTCAACGGAAAACCTGTTT
IFNGR	TCACTGTCCTCAAACGTG
*3'RACE*	
omOLIB32ALL	GTGAACAACCGTCCAAATGGCTCA
	
**Zebrafish**	
***3'RACE***	
zftrall1S	TGTGGACACAGTTACTGTATGAGCTG
zftrall2S	TGCAGACAGACCTTCACTCCAAGACC

### Cloning and sequencing of PCR products

The trout PCR products were purified with Sephacryl S-400 columns (Pharmacia, Paris, France) and then cloned using the TOPO T/A Cloning Kit (Invitrogen). Upon transformation of *E. coli*, plasmid was isolated by the Nucleospin Plasmid Quickpure kit (Nucleospin; Macherey-Nagel, Düren, France). Purified plasmids were subjected to automated sequencing with M13 direct and reverse primers and with internal primers for long transcripts. Zebrafish RACE products were treated in a comparable manner but with different reagents: PCR products were purified on Qiaquick spin columns (Qiagen, Courtaboeuf, France) and cloned in the pGEMT-easy vector (Promega, Charbonnières-les-Bains, France); plasmids were purified with QiaPrep Miniprep kits (Qiagen) and sequencing was performed initially with SP6 and T7 primers.

### Real-time RT-PCR assay

Real-time RT-PCR reactions were performed using the SYBR green reagent from Applied Biosystems (Applied Biosystems, Les Ulis, France) and Eppendorf Mastercycler realplex2 S (Eppendorf, Le Pecq, France), according to manufacturer's instructions. All reactions were performed in duplicate. Data analysis was performed as described in the ABI PRISM 7700 sequence detection bulletin #2 from Applied Biosystems, following the Δ Ct method. Oligonucleotides used for real-time RT-PCR are indicated in Table 4.

### Strategy for identification and alignment of *finTRIM *sequences

The finTRIM sequences were assembled using the tools of the Genetic Computer Group (GCG; Madison University, Wisconsin) and the DNA strider software (CEA, Gif-sur-Yvette, France). The sequences were subjected to multiple sequence alignments using GCG (pileup) or ClustalW, and multiple alignments were edited using Boxshade software. Systematic searches for finTRIM were done using the tblastn program with rainbow trout or zebrafish sequences as a query, on available EST and genome databases. Searches in EST databases were mainly performed at the NCBI and Dana Farber Institute websites. Blast queries on complete genomes were sent to Ensembl and NCBI. The last assembly of the zebrafish genome (zv7) was searched at the Ensembl site. When relevant genomic regions were identified, potential exons were manually identified by comparison with known sequences, notably RACE clones, with the help of predictions made by Genscan .

### Detection of positive selection

The dataset for positive selection analysis was prepared from the zebrafish group A finTRIM (ftr) sequences that were found on the Ensembl zebrafish Zv7 assembly. Three datasets were prepared, corresponding to sequences coding for the RING and B-box 1 and 2 (RBB) domains (55 sequences) and the B30.2 domain (38 sequences). Domains were identified by the web-based tool Simple Modular Architecture Research Tool (SMART) at . A multiple sequence alignment was made for each domain with ClustalW within the *MEGA*4 software and gaps were removed from the alignment. The final datasets consisted of 155 codons for the RING and B-box domains and 145 codons for the B30.2 domain. The phylogenetic trees were constructed by the Neighbor-Joining method using M*EGA*4.

The Codeml program of the Phylogeny Analysis by Maximum Likelihood (PAML) package [63], retrieved from , was used for the detection of positive selection. The models M0, M1a, M2a, M7 and M8 were employed. The ratio of synonymous (*dS*) to non-synonymous (*dN*) substitution rates, ω = *dS*/*dN*, is determined by the program. A value of ω < 1 indicates negative, purifying selection, ω = 1 indicates neutral evolution and ω >1 indicates the occurrence of diversifying, positive selection. We used the site-specific model that allows ω to vary among sites. The null models M0, M1a and M7 do not allow the existence of positively selected sites (ω >1), while the alternate models M2a and M8 allow ω >1. M8 follows a beta(*p*, *q*)-distribution and is less stringent than M2a. Within the models, a Maximum Likelihood algorithm is used, whereby the sites are allocated under classes of different ω probabilities. Sites allocated under the class with ω >1 are considered as being under positive selection and were identified by a Bayes Empirical Bayes (BEB) analysis. Significance of outcome was confirmed by a likelihood ratio test (LRT). In the LTR we took twice the difference in log likelihood (2ΔlnL) between the nested models and used the chi-square test with the degrees of freedom (df) being the difference in free parameters between the two models (M1a vs. M2a and M7 vs. M8). Tests were considered positive when *p *< 0.001. Sites identified by BEB with a posterior probability higher than 95% were considered significant.

### Analysis for recombination

To test for interference of recombination on the PAML results, we implemented a test by the algorithm PARRIS [64]. Under PARRIS, the PAML models M1a-M2a are employed with incorporation of site-to-site variation in synonymous substitutions rates and partitioning of data. We used the codon model for evolution GY94 × HKY85 and a discrete distribution of three bins for synonymous and for non-synonymous rates. Significance of results was tested by a LRT.

We detected recombination breakpoints by the algorithm GARD [65]. We used the HKY85 model with general discrete distribution of rates across sites. We performed two screenings, for 2 or 20 breakpoints. The detection was validated by corrected Akaike's information criterium (c-AIC) for best-fitted model selection. Both PARRIS and GARD are integrated in the HyPhy software package that was retrieved from .

### Nucleotide sequence accession numbers

Sequences of rainbow trout and zebrafish finTRIM experimentally produced have been deposited in the EMBL database under accession no AM887792–AM887863 (rainbow trout) and AM941305–AM941371 (zebrafish).

## Authors' contributions

LVDA, JPL, MY, PB conceived and designed the experiments. LVDA, JPL, MY, EL, VB and PB performed the experiments. LVDA, JPL, MY, EL, VB and PB designed the figures. LVDA, JPL, MY, PH, AB and PB undertook the data analysis. LVDA, JPL, PH, AB and PB prepared the manuscript. All authors have read and approved the final manuscript.

## Supplementary Material

Additional file 1 **Figure S1 – Distance trees of RBB (A) or B30.2 (B) domains from zebrafish ftrs.** Sequences have been aligned using Clustalw, and the distance tree built using a NJ algorithm. Click here for file

Additional file 2 **Table S1.** Details about zebrafish finTRIM-related genes: nomenclature, location, accession numbers. Click here for file

Additional file 3 **Table S2.***ftr*-like genes in other teleosts: genes corresponding to significant blastp hits on Ensembl ab initio databases for medaka, stickleback and tetraodon are reported: Ensembl name, location, number of exons. Click here for file

Additional file 4 **Figure S2.***ftr *sequences used for PAML analysis. The alignments of nucleotide and protein sequences used for the positive selection analysis are reported for RBB (A) and B30.2 (B) domains. Click here for file

Additional file 5 **Table S3.** Results of GARD analysis. Click here for file

Additional file 6 **Table S4.** PAML results of zebrafish finTRIM groupA sequences: detected sites under positive selection by BEB analysis. Section B30.2: PAML analysis was carried out with both the complete B30.2 domain and the segmented alignments before (1 to 285 nt) or after (286 to 485 nt) the detected breakage point by GARD. Section RBB : PAML analysis was carried out with both the complete RBB domain and the segmented alignments before (1 to234 nt) or after (238 to 465 nt) the detected breakage point by GARD (section1). The numbering of amino acids corresponds with the sequence of zebrafish finTRIM 13. Click here for file
