# Supplementary figures and images for: A large new subset of TRIM genes highly diversified by duplication and positive selection in teleost fish
Source: BMC Biol. 2009 Feb 5;7:7. doi: 10.1186/1741-7007-7-7 (PMC2657112; doi:10.1186/1741-7007-7-7)

Fig. S1A - tree based on RBB domains

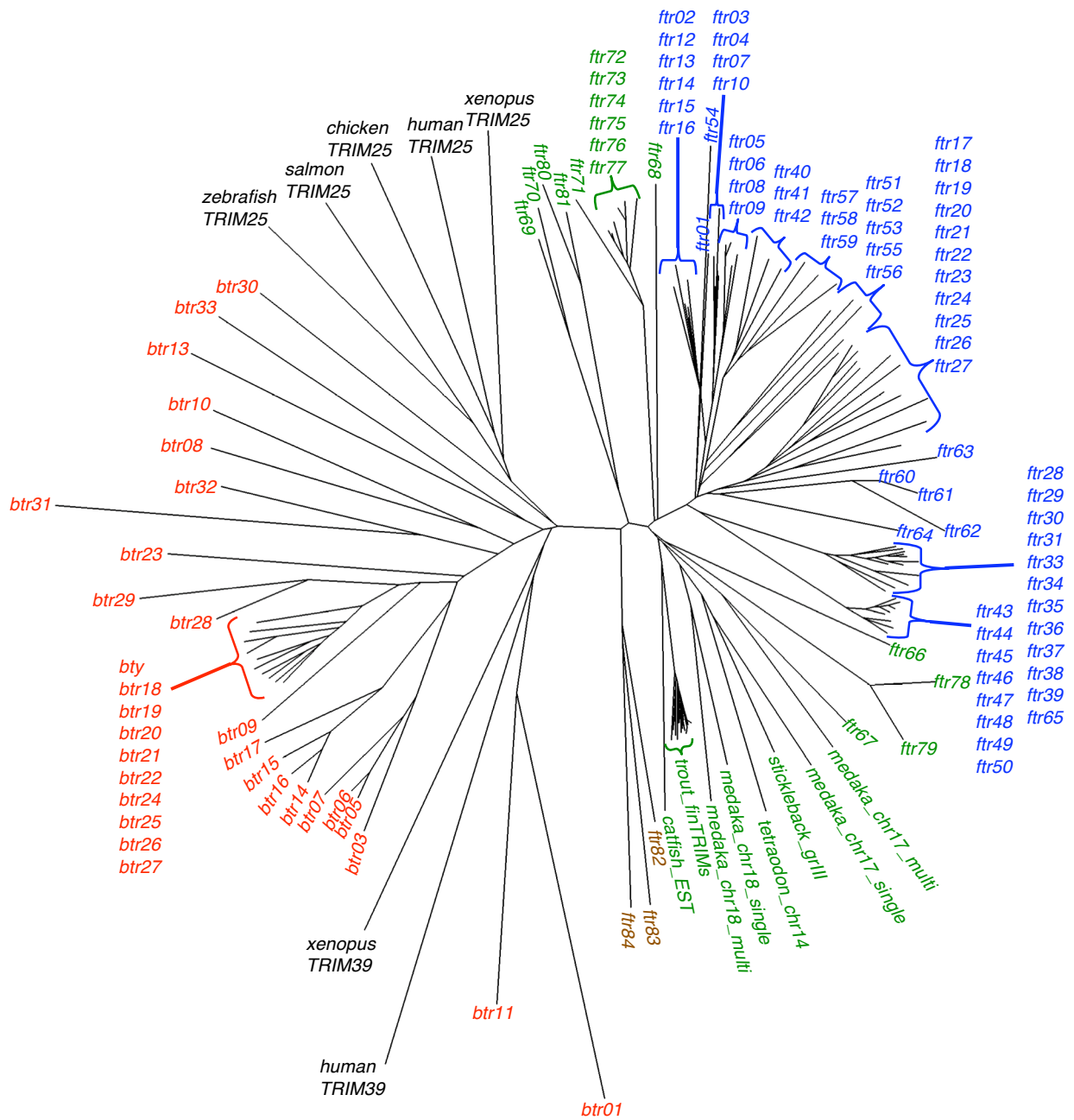

**Fig. S1B - tree based on B30.2 domains**

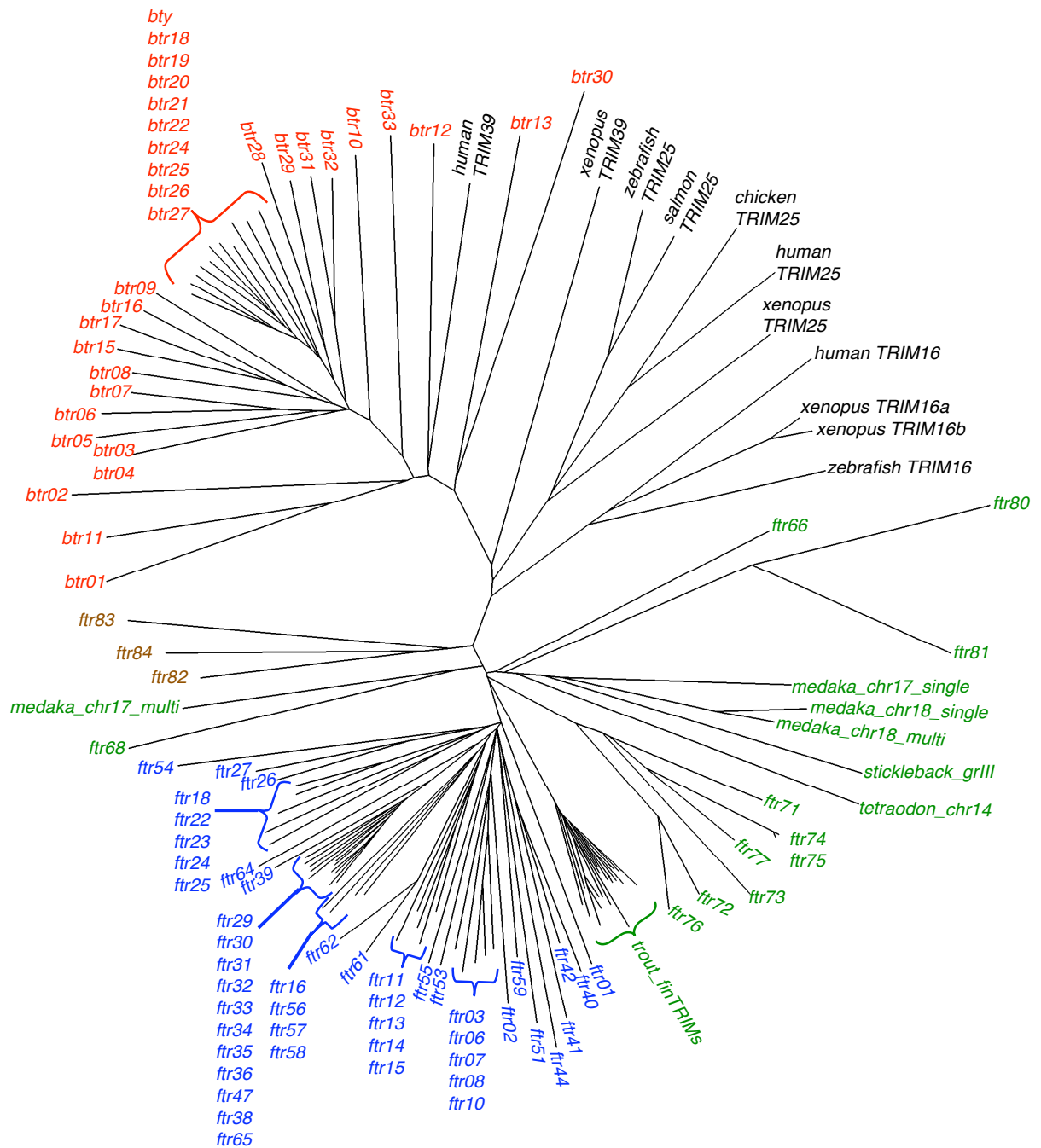

Supplement: Additional file 1 — Figure S1 – Distance trees of RBB (A) or B30.2 (B) domains from zebrafish ftrs. Sequences have been aligned using Clustalw, and the distance tree built using a NJ algorithm. [file 1741-7007-7-7-S1.pdf]
