# Supplementary material for: A large new subset of TRIM genes highly diversified by duplication and positive selection in teleost fish
Source: BMC Biol. 2009 Feb 5;7:7. doi: 10.1186/1741-7007-7-7 (PMC2657112; doi:10.1186/1741-7007-7-7)
Supplement: Additional file 3 — Table S2.ftr-like genes in other teleosts: genes corresponding to significant blastp hits on Ensembl ab initio databases for medaka, stickleback and tetraodon are reported: Ensembl name, location, number of exons. [file 1741-7007-7-7-S3.pdf]

Supplementary table S2 : ftr -like genes in other teleosts.

Significant blastp hits from Ab initio databases associated to genome sequences of medaka, stickleback and tetraodon at Ensembl.

The ensembl name of the hits and the location on the chromosome are indicated

Medaka

Name	Name	Start (AUG)	End (stop)	Nb of exons
GENSCAN00000110958	Chr:1	34177110	34174613	6
GENSCAN00000036476	Chr:14	22419892	22424198	6
GENSCAN00000093499	Chr:14	22413866	22410525	7
GENSCAN00000025963	Chr:14	2439654	2442003	1
GENSCAN00000091525	Chr:15	21564724	21562240	6
GENSCAN00000018074	Chr:17	11616787	11618454	1
GENSCAN00000045821	Chr:17	30120311	30122866	4
GENSCAN00000108815	Chr:17	8244213	8245700	1
GENSCAN00000018500	Chr:17	8269326	8270972	1
GENSCAN00000070019	Chr:17	8279510	8277849	1
GENSCAN00000018504	Chr:17	8262618	8260954	1
GENSCAN00000097849	Chr:17	8965648	8967166	1
GENSCAN00000047786	Chr:17	8631857	8630240	1
GENSCAN00000057649	Chr:17	10036207	10034605	1
GENSCAN00000073091	Chr:17	10068191	10066680	1
GENSCAN00000032774	Chr:17	9045824	9047394	1
GENSCAN00000022620	Chr:17	483769	482269	1
GENSCAN00000022520	Chr:17	445369	444092	1
GENSCAN00000069132	Chr:17	8412274	8410262	1
GENSCAN00000054450	Chr:17	8852669	8851349	1
GENSCAN00000045819	Chr:17	30125876	30132497	6
GENSCAN00000035461	Chr:17	8231476	8233143	1
GENSCAN00000085752	Chr:17	10081045	10079334	1
GENSCAN00000086075	Chr:17	9489929	9491139	1
GENSCAN00000104494	Chr:17	9475337	9476887	1
GENSCAN00000055448	Chr:17	9074171	9075448	1
GENSCAN00000055706	Chr:17	8250312	8251708	1
GENSCAN00000075396	Chr:17	8703396	8704995	1
GENSCAN00000018446	Chr:17	10046106	10044564	1
ENSORLG00000006248	Chr :17	8833339	8833350	1

GENSCAN00000071710	Chr :17	9100254	9101691	1
GENSCAN00000112517	Chr :17	10013153	10014770	1
GENSCAN00000056194	Chr :17	9564886	9563350	1
ENSORLG00000006437	Chr :17	9637113	9635985	1
GENSCAN00000117671	Chr:18	2254254	2252608	1
GENSCAN00000029902	Chr:18	2688084	2689760	1
GENSCAN00000005659	Chr:18	2868240	2870009	1
GENSCAN00000095092	Chr:18	2406731	2405049	1
GENSCAN00000021057	Chr:18	29403660	29402189	1
GENSCAN00000004320	Chr:18	25058223	25059432	1
GENSCAN00000113407	Chr:18	28915816	28916568	1
GENSCAN00000025515	Chr:18	26859049	26859060	2
GENSCAN00000066497	Chr:18	2708259	2709922	1
ENSORLG00000010802	Chr :18	26846784	26847049	1
ENSORLG00000010794	Chr :18	26721724	26719292	1 or 2
GENSCAN00000105092	Chr :18	26875201	26876164	1
GENSCAN00000116846	Chr :18	2692271	2693430	1
GENSCAN00000070512	Chr :18	2436609	2437958	1
GENSCAN00000070508	Chr :18	2439665	2441302	1
GENSCAN00000115691	Chr:2	23838584	23845615	6
GENSCAN00000079805	Chr:21	13166342	13167378	1
GENSCAN00000062051	Chr:24	23288265	23286613	1
GENSCAN00000047149	Chr:5	11676226	11674517	1
GENSCAN00000032159	Chr:6	26441168	26442661	1
GENSCAN00000059700	Chr:6	26487403	26493807	5
GENSCAN00000015195	Scaffold1875	3213	3822	1
GENSCAN00000070218	Scaffold2084	11300	12958	1
GENSCAN00000065517	Ultracontig90	2034459	2035181	1
GENSCAN00000053909	Scaffold877	53000	54058	1
GENSCAN00000027868	Ultracontig236	2139973	2142153	1
GENSCAN00000058260	Scaffold3348	3960	5604	1
GENSCAN00000058503	Scaffold3544	2917	1674	1
GENSCAN00000116323	Ultracontig218	161435	160647	1
GENSCAN00000107211	scaffold785	~50000		1
GENSCAN00000020147	Scaffold3061	4013	2406	3+
GENSCAN00000044369	scaffold1708	~16000		1
GENSCAN00000019337	scaffold2982	3168	2624	1

GENSCAN00000060761	Scaffold717	92332	93006	1
GENSCAN00000062026	scaffold4570	3439	4332	1
GENSCAN00000100797	Scaffold907	58556	57782	1
GENSCAN00000034217	scaffold5209	3344	3775	1
GENSCAN00000086620	scaffold3964	630	<1	1 ?
GENSCAN00000122477	Scaffold757	~56000	?	1
GENSCAN00000108983	scaffold5344	2934	1860	1
GENSCAN00000117118	Scaffold1690	9731	10933	1
GENSCAN00000051507	Scaffold510	~39000		?
GENSCAN00000075770	Scaffold4611	5113	>5436	?
GENSCAN00000063126	Scaffold2318	~8000	?	?
GENSCAN00000090553	scaffold3635	~2000	?	?

Stickleback

Subject Name	Chromosome Name	Start	End
GENSCAN00000022585	Chr:groupIII	14324861	14325445
GENSCAN00000036462	Chr:groupIII	14109694	14110287
GENSCAN00000023536	Chr:groupIII	13188065	13188691
GENSCAN00000030816	Chr:groupIII	14269828	14270412
GENSCAN00000039642	Chr:groupVII	1860109	1861302
GENSCAN00000039635	Chr:groupVII	1872250	1873691
GENSCAN00000039669	Chr:groupVII	14230586	14232343
GENSCAN00000019900	No data		

Tetraodon

Subject Name	Chromosome Name	Start	End
GIDT00016540001	Chr:14	9469225	9469803
GIDT00014255001	Chr:17	5530066	5530290
GIDT00020308001	Chr:18	1067127	1067760
GIDT00025424001	Chr:18	9779431	9779721
GIDT00016155001	Chr:3	3889493	3893052
GIDT00014126001	Chr:7	1604391	1605994
GIDT00014125001	Chr:7	1609252	1610292
GIDT00021469001	Chr:9	2193382	2193681
GIDT00025216001	Un_random	16652089	16652679
