# Supplementary material for: A large new subset of TRIM genes highly diversified by duplication and positive selection in teleost fish
Source: BMC Biol. 2009 Feb 5;7:7. doi: 10.1186/1741-7007-7-7 (PMC2657112; doi:10.1186/1741-7007-7-7)
Supplement: Additional file 5 — Table S3. Results of GARD analysis. [file 1741-7007-7-7-S5.pdf]

Table S3. Results of GARD analysis.

domain	search nr sites	c-AIC BP0	c-AIC BP1	delta c-AIC	position	lnL
B30.2	2	15941.80	15898.9	42.96	285	-7874,11
	20	15870.80	15842.1	28.67	285	-7819,98
RBB	2	17847.00	17592.3	254.63	236	-8773,79
	20	17808.60	17545.4	263.22	236	-8732,86

The c-AIC, Akaike`s information criterium indicates which model fits the data best.
The positions of detected breakage points correspond with the nucleotide position in the alignment that we used for the GARD analysis. They correspond with the $Valine_{484}$ of zebrafish finTRIM13 in the B30.2 domain and $Glycine_{107}$ in the RBB domain
